# Barriers and facilitators of physical activity among Latina immigrant and Mexican mothers living in the US and Mexico: A qualitative study

**DOI:** 10.1371/journal.pone.0290227

**Published:** 2023-08-31

**Authors:** Nancy Jacquelyn Pérez-Flores, María Pineros-Leano, Katherine Damian, Ashley M. Toney, Liliana Aguayo

**Affiliations:** 1 Brown School of Social Work, Washington University in St. Louis, St. Louis, Missouri, United States of America; 2 School of Social Work, Boston College, Chestnut Hill, Massachusetts, United States of America; 3 Department of Psychology, University of Illinois at Urbana-Champaign, Champaign, Illinois, United States of America; 4 Department of Cardiovascular and Metabolic Sciences, Cleveland Clinic Lerner Research Institute, Cleveland, Ohio, United States of America; 5 Hubert Department of Global Health, Emory University, Atlanta, Georgia, United States of America; Caleb University, NIGERIA

## Abstract

Latina immigrant women are among the least physically active when compared with women in other racial/ethnic groups in the US. Similarly, Mexican mothers in Mexico have low rates of physical activity. Motherhood and immigration experiences are recognized barriers to engage in physical activity among Latina immigrant mothers. Less is known about the factors that promote and limit physical activity engagement among Mexican mothers in Mexico, and how their experiences compare with their immigrant counterparts. This transnational qualitative study aimed to investigate the barriers and facilitators of physical activity of 25 Latina mothers in Mexico and the US. Low-income Mexican mothers of kindergarten aged children and Latino mothers of similar aged children were recruited in San Luis Potosí, Mexico and central Illinois, US. Semi-structured interviews were administered by two bilingual and bicultural researchers in participants language of preference. Interviews were transcribed verbatim and analyzed using a thematic network approach and multi-stage coding analysis guided by the Socio-Ecological Model framework. We found that at the macro-level: 1) familial obligations, and 2) cold weather after migrating; at the mezzo-level: 1) changes in walking patterns, and 2) social cohesion (e.g., lack of an invitation to engage in activities); and at the micro-level: 1) individual perceptions, particularly unattainable perceptions of physical activity and 2) shift exhaustion were perceived as barriers and occasionally facilitators of physical activity by mothers in both countries. Context-specific interventions are needed to increase women’s physical activity levels in the US and Mexico.

## Introduction

Physical activity offers numerous health benefits, including the reduction of chronic disease risks [1] improved cognitive functioning [2], and lower levels of depression and anxiety [3]. However, less than 25% of adults report meeting the physical activity recommendations in the US [2, 4]. Latina women have lower rates of physical activity than any other ethnic/racial group in the US [5]. Similarly, in Mexico, physical inactivity is one of the major contributors to the increasing prevalence of coronary heart disease and type 2 diabetes, one of the leading causes of death in the country [6]. When compared with Mexican men, Mexican women have a higher prevalence of physical inactivity [6]. In a national study conducted in 2021, 46.7% of men reported being physically active compared with 33.3% of Mexican women, who reported the lowest percentage since 2013 [7]. Promoting physical activity among Latina immigrant and Mexican women, particularly among mothers, is highly important as previous studies have suggested that children’s physical activity levels mirror those of their parents [8].

Previous studies have identified several barriers to engaging in physical activity among Latina mothers, including caregiving responsibilities [9], lack of childcare [10], lack of exercise partners [11], disapproval from others [12], and high level of acculturation to the US culture [13, 14]. Redfield and colleagues (1936) define acculturation as “when groups of individuals having different cultures come into continuous first-hand contact, with subsequent changes in the original cultural patterns of either or both groups” [15 p 149]. Acculturation is a multifaceted process in which recent immigrants interact with the dominant culture and alter their actions, attitudes, and beliefs [16].

The association of acculturation and physical activity has been mixed. It is unclear how activity levels of immigrant women compare to their non-immigrant counterparts [17]. For example, Benitez and colleagues’ (2016) integrative review of literature on studies that examined acculturation as it relates to physical activity in Latinas in the U.S, found 33 studies that yield inconsistent measurement and conceptualization of acculturation and physical activity across studies [17]. However, a study of 51 Latina immigrant mothers found that women perceived that their physical activity levels decreased after migrating to the U.S. [13]. Some of the reasons cited included colder weather, more frequent driving, and less strenuous work in the US [13]. Another study of Latina immigrant mothers found that time constraints, weather, lack of access to recreational facilities, the cost of sports equipment, and neighborhood safety limit physical activity engagement [14]. These studies indicate that Latina immigrant women encounter several barriers when trying to engage in physical activity.

Despite the extensive literature documenting the influence of acculturation [18] to the US to physical inactivity, transnational studies of the experiences and barriers that Latina mothers face in the US and how these compare to those of Mexican women, are lacking. Translational studies may help better understand how acculturation influences physical activity by elucidating the different barriers and facilitators Latina immigrant and Mexican mothers face when engaging in physical activity in their country after immigration as compared to their counterparts in their country of origin (i.e., Mexico).

The Socio-Ecological Model was utilized as a guiding framework to understand the complex perceptions of Latina immigrant and Mexican mothers regarding physical activity. The Socio-Ecological Model suggests that people’s health behaviors are influenced by multiple levels ranging from the micro-system to the macro-system [19]. The micro-system includes intrapersonal and interpersonal factors, the mezzo-system consists of institutional and community characteristics, and the macro-system consists of larger societal and political factors [19, 20]. To this end, the Socio-Ecological Model provides a lens to better understand the competing factors influencing Latina mothers’ decisions around physical activity situated within their contextualized environment.

A better understanding of the barriers and facilitators that Latina immigrant women and Mexican women encounter when engaging in physical activity can support the development of more effective, context-specific, and culturally sensitive physical activity interventions. In this transnational study, the authors aimed to address this critical gap by identifying and comparing the perceptions and experiences of physical activity among Latina immigrant women residing in the US and Mexican women and Mexico, all of whom were mothers. The Socio-Ecological Model was utilized as a guiding framework to understand the complex perceptions of Latina immigrant and Mexican women regarding physical activity.

## Methods

The authors conducted a qualitative, cross-sectional study called the Holistic Obesity Prevention Study (HOPS). HOPS was a binational study designed to investigate factors influencing Latina immigrant and Mexican mothers and their preschool-aged children’s knowledge, attitudes, and beliefs regarding obesity through semi-structured interviews. Data were collected from Latina immigrant and Mexican women living in Mexico and the US (N = 25). In Mexico, data were collected from January to February 2016. In the US, data were collected from February to April 2016.

### Recruitment and eligibility

The recruitment efforts targeted low-income women living in Mexico, and in the US. Women were eligible to participate if they: a) were 18 years of age or older, b) self-identified as Mexican or Latina, c) spoke English or Spanish, d) had a 4- to 6-year-old child living in the same household, and e) had no physical or medical condition that would affect their participation or ability to perform physical activity (e.g., cognitive, or physical disability). In Mexico, recruitment flyers were distributed among all women who had a child attending a kindergarten located in a low-income, semi-urban neighborhood in the outskirts of San Luis Potosi, SLP. In the US, recruitment materials were posted in local community centers, public parks, laundromats, and local churches across several semi-urban cities in Central Illinois. Participants in the US were also recruited using snowball sampling strategies and referrals from community leaders.

### Data collection procedures

Data were collected through a two-hour meeting where trained researchers (LA and MPL) first read and explained the consent to the potential participants. As part of the inform consent process participants were informed that their participation was voluntary and they could refuse to participate or terminate the interview. Once women provided written informed consent and accepted to participate, they filled out a demographic information survey. The interviews were conducted in the participant’s language of preference (English or Spanish). The semi-structured interview guide was designed and pilot tested by a transdisciplinary team with combined expertise in public health, human development, nutrition, and social work. Field notes were taken during the interview to document any departure from the protocol (e.g., participant not interviewed in a private setting due to children or others interrupting). The researchers who collected data are bilingual and bicultural. They were completing their doctoral studies when the study was conducted. On average, interviews lasted one hour to complete but they ranged from 40 to 80 minutes. Standardized probes were used to expand and clarify women’s responses. Of the 25 interviews completed, 24 were conducted in Spanish and one in English. All interviews in Mexico (n = 12) were conducted at a private office in the kindergarten’s library. In the US, most interviews were conducted in a private room at participants’ homes, and one was completed at a private office in a local church (n = 13). The mother and child were usually the ones present during the interview. In few instances, there were more people present during the interview, but they did not participate. None of the mothers who volunteered to participate refused to do so after their appointments were scheduled and none terminated the interview prior to completion.

Women in both Mexico and the US were compensated $20 US dollars (equivalent to $400 Mexican pesos in 2016) for participating in the study. The study received institutional review board (IRB) approval from the University of Illinois at Urbana-Champaign (IRB Approval # 16113), the Kindergarten’s Principal in Mexico, and the Secretary of Education of the Government of the State of San Luis Potosi, Mexico (Secretaría de Educación de Gobierno del Estado, SEGE).

### Data analysis

All interviews were recorded and transcribed verbatim and analyzed by a team of bilingual and bicultural researchers in the interview language. Data saturation was reached independently for the group in Mexico and U.S. Descriptive statistics were calculated and reported overall and by country of residence. Data were analyzed using a thematic network analysis following a multi-stage approach [21]. First, two coders [NJFP and KD] read all the interviews separately and identified general codes. The coders then met with the principal investigators [LA and MPL] and developed a codebook by going through the different codes they identified and defining each code. Once the codebook was developed, the coders recoded all the interviews and identified relevant quotes. To ensure the trustworthiness of the data, all coders [NJPF & KD] reached at least an 80% interrater reliability agreement. Any codes that did not meet the 80% agreement were further discussed until agreement was reached. NJPF and KD organized the codes into the levels of the Socio-Ecological Model by using the Nvivo 12. The authors identified that the macro level refers to environmental and societal influences (e.g., familial obligations), the mezzo level to intrapersonal influences (e.g., the impact of social cohesion), and the micro level to factors at the individual level (e.g., shift exhaustion). A three-step process was followed to ensure the linguistic accuracy of the translated quotes. The first step was to translate the quotes into English, then into Spanish, and lastly back to English.

## Results

### Sample description

Of the 25 women who participated in the study, 23 were born in Mexico, and 2 were born in Central America (i.e., Costa Rica and Guatemala). Twelve lived in Mexico, and 13 lived in Central Illinois. The average age of women was 32.5±6.4 years. All participants were fluent in Spanish, and most had less than a high school education (n = 16, 64%). For women living in the US, the average time spent in the US was 13.7±5.4 years. The mean women’s body mass index (BMI) was 30.3±9.1 kg/m^2^ with women’s weight status ranging from underweight (n = 1) to severe obesity (n = 5).

#### Codes

Latina immigrant and Mexican women held different perceptions of physical activity that aligned with each level of the Socio-Ecological Model. We identified two codes that explained their engagement with physical activity within each level. At the macro level, we found: 1) familial obligations and 2) cold weather perceptions after migrating to the US hindered their ability to engage in physical activity. At the mezzo level, we found: 1) changes in modes of transportation after migration and 2) the influence of social cohesion. Lastly, at the micro-level, we found: 1) expectations of physical activity and 2) exhaustion from paid employment Table 1.

**Table 1:** Key codes and illustrative quotes.

Macro-level codes	
*Familial obligations*	“Not having enough time, not having childcare help. A lot of times, if you do not have gym memberships, which are expensive, you have to watch your own children while you work out and it’s hard to work out while the kids are tugging at your legs. Or asking you what 11 plus 10 is. So, it can be difficult to have a really good workout.”—Araceli, US
*Cold weather perceptions after migrating to the US*	“…since it was hot [in Mexico] they [my parents] always walked but here since it’s cold in winter they don’t come out until summer, maybe that’s why they suffer more in the winter as they don’t come out the same because gyms are expensive and because there in the field, nobody charges you for walking.”—Laura, US “Well, a big thing is weather because it’s always cold here. And as a parent, I see it differently, like nope, it is too cold outside. As opposed to when I was a kid, and I was out there with no jacket in the middle of winter. It’s a different side of the coin, I guess.”—Norma, US
Mezzo-level codes	
*Changes in modes of transportation after migration*	“…there [in Mexico] you walk too much; it is not like here [the US] that you get off the car and you are already at the door of the supermarket […]. Now when I go there [to Mexico], we lose weight because you walk a lot, a lot. You walk too much […], you walk everywhere to take the bus. You walk about 10 to 15 minutes to go to the supermarket [because] the car is very far away from where you park your car to enter the supermarket. Over there, there is a lot of physical activity; more than anything, you walk too much.”—Norberta, US “They […]didn’t care much about it, we walked a lot because we didn’t have a vehicle to transport ourselves with, so they always liked to walk a lot. They would tell us that instead of taking the bus or something like that, it was going to be walking or cycling.”—Marceliana, MX
*The influence of social cohesion*.	“… I try to take my children to the park, for example, to run, to play, to exercise a little. Here we are a bit locked up like right now. It is a bit cold, [so] I try to take them to a store for a walk at least when we go shopping, and we all go as we cannot walk outside, so we walk around in the store, and well that is the way for us to exercise a little.”—Norma, US
Micro-level codes	
*Expectations of physical activity*	“Well, the truth is that I do not have much information, because I have not practiced it or know more or less, I have been told that it is very good to relax and so on, but the truth is I could not tell you because I have not had the experience…sometimes I don’t have the information, but I feel like it’s because I don’t know the costs, sometimes you think it’s not very expensive…”—Abigail, MX “I don’t like it, Well I don’t like what I see, I once practiced Pilates, and it gave me the impression that it was similar, I don’t have much flexibility in my body, so I struggled a lot with Pilates, and like yoga I saw it very similar, and rather I like more movement to feel like I’m sweating, yoga gives me the impression that it annoys me like I see them and tell them to move and do something, that’s my perception because I’ve never lived it [Laughs]I don’t feel like getting close.”—Ximena, US
*Shift Exhaustion*.	“Only she (daughter) engages in physical activity (e.g., jumping). I tell her that I will watch [her] (laughs). There are times that I do work out with her, but there are times I work in the afternoon every day, and well, I come back somewhat tired, to be honest.”—Julia, MX

#### Macro-level

*Familial obligations*. Most women in Mexico and the US mentioned they do not prioritize physical activity because their limited time and energy is spent doing house chores (e.g., laundry) and working. Women mentioned having no childcare to engage in physical activities and having family obligations occupy most of their time. When one participant was asked why there was no chance for her to engage in physical activity, she responded, “For doing other things, due to doing the laundry, for doing the chores of the house, to be working.”—Julia, MX.

*Cold weather perceptions after migrating to the US*. Cold weather was identified as a barrier to physical activity for all women (n = 13) who migrated in the U.S., where the temperature is frequently below 32 degrees Fahrenheit or 0 degrees Celsius. When one participant was asked why there is limited engagement in physical activity, she responded, “…since it was hot [in Mexico] they [my parents] always walked but here since it’s cold in winter they don’t come out until summer, maybe that’s why they suffer more in the winter as they don’t come out the same because gyms are expensive and because there in the field, nobody charges you for walking.”—Laura, US.

#### Mezzo-level

*Changes in modes of transportation after migration*. Women attributed changes in modes of transportation after migration from walking to using cars as their most reliable form of transportation. Women in the US (n = 9) mentioned that they did not have accessible transportation in Mexico, so they would rely heavily on walking as their primary method of transportation. However, after migrating to the US, long-distance commutes to stores or markets mainly required them to depend on cars as their form of consistent transportation. In contrast, women living in Mexico mentioned that even if they had access to transportation (e.g., bus, car), they prefer to walk or ride their bike due to having an array of stores and/or markets nearby.

*The influence of social cohesion*. Some women cited a lack of an invitation as a reason for not participating in physical activity, suggesting having company could encourage participation in physical activity. When a participant was asked why they did not participate in physical activity they responded, “Well, note that I have not done it because no one has invited me; I do not know people who say, ‘I am going to yoga, are you interested in going?’—Sophia, US.

#### Micro-level

*Expectations of physical activity*. Most women (n = 20) in the US and Mexico felt limited by their lack of knowledge of physical activity, perceived high-price and cost-prohibited, lack of experience with different types of physical activity (e.g., yoga), and their expectations of what constitutes physical activity (e.g., sweating excessively). Twenty mothers reported a sense of accomplishment through sweating after working out.

*Shift exhaustion*. Six women from both the US and Mexico mentioned coming home tired from long work hours, limiting their time to engage in physical activity. One mother mentioned: “Well, since I worked for a while, of course, I got really tired because I walked a long, long time there in [Mexico], I would go up and downstairs and I would get very tired.”—Julie, US

## Discussion

The objective of this study was to identify the barriers and facilitators of physical activity at the macro, mezzo, and micro levels, as perceived by Latina immigrant women and Mexican women. We found that at the macro level: 1) familial obligations and 2) cold weather perceptions after migrating to the US; at the mezzo level: 1) changes in modes of transportation after migration and 2) the influence of social cohesion; and lastly, at the micro level: 1) expectations of physical activity and 2) shift exhaustion influenced Latina immigrant and Mexican mother’s physical activity engagement.

### Macro-level findings

We found that women in the US and Mexico reported low levels of physical activity due to the chores they prioritized at home, which is consistent with values of *familismo* and *marianismo* in Latino culture [22]. In a previous study that contrasted the perceptions of Anglo American and Mexican American women the authors also noted that Mexican American women prioritize their families’ needs over their own [23]. Women direct their daily activities primarily to taking care of their children and house chores, which are activities that are complex to quantify [20]. To increase physical activity levels amongst Latina and Mexican women, there needs to be accessible information that is culturally and linguistically grounded in Spanish, reframing family values such as *marianismo* and *familismo*, and familial and individual goals [24]. For instance, it is important that recommendations acknowledge the role of *marianismo*, while emphasizing that to provide the best care to children, it is necessary to take care of oneself.

We also found that the cold weather post-migration impacted physical activity, which mirrors previous findings that indicate that environmental influences can be a barrier to physical activity [25]. We identified cold weather as a barrier to physical activity among Mexican women born in Mexico, but once they moved to the U.S, they identified cold weather as a barrier due to immigration to an unfamiliar climate. These findings indicate that it is essential to provide accessible and affordable places where Mexican women and their families can engage in physical activity during the cold months of winter and heat in the summer. For example, encouraging mothers to continue accessing free indoor spaces, such as the mall, can increase cardio levels and maintain them active [26].

### Mezzo-level findings

#### The influence of social cohesion

Family, friends, children, and other broader social networks play an essential role in facilitating or discouraging Latina women’s physical activity participation. Participants in the study also shared that they were active when walking their children to school, church, parks, and shopping centers. Parental transportation to places to be active has consistently been associated with their child’s physical activity level [27, 28]. Both parents and their children are ecological influences on each other’s physical activity patterns. These findings agree with previous literature that has found that Latinas who know people who exercise or belong to a community or faith-based group are more likely to be physically active [5]. Given the role that social influences seem to have on physical activity [27, 28] future research should continue investigating the dual facilitation of physical activity through family dynamics. Further, efforts to increase physical activity among Latina and Mexican women should consider shifting from individual-focus to family or group-based approaches.

#### Changes in modes of transportation after migration

We found that the US-residing women relied heavily on driving as their method of transportation. Women living in Mexico preferred walking or bike riding even when public transportation was available. However, in the US, women’s walking frequency was reduced due to increased travel distance necessitating car use. These results indicate that Latina immigrant women in the US have acculturated by more frequently utilizing cars as a form of transportation instead of walking. More qualitative information on neighborhood factors that promote or discourage walking among Latino immigrant families is needed to understand the changes in walking patterns after migration. While most Latino walkability literature is focused on obesity and neighborhood walkability [1, 29, 30], the authors identify socio-ecological patterns of walkability for physical activity.

### Micro-level findings

#### Expectations of physical activity

Latina women have reported that a lack of time and being too tired has contributed to their lack of physical activity in previous research [31]. We expand the literature recognizing that US- and Mexico-based women felt their familiarity with physical activity is limited due to lack of understanding of associated costs (or financial barriers), inexperience with different types of activity, and differing definitions and unattainable expectations of what constitutes physical activity. Our findings are consistent with previous literature noting that lack of knowledge and economic limitations were barriers for Latina women’s physical activity [10, 23, 32]. Culturally-competent efforts to promote physical activity need to encompass various expectations and experiences of physical activity. Based on our findings, Latina and Mexican mothers will benefit from approaches that promote small changes, and highlight that even small increases in physical activity yield meaningful health benefits for currently inactive people.

#### Exhaustion from paid employment

The authors found that women living in Mexico and the US reported exhaustion after work, which resulted in insufficient time for physical activity. Parents that migrate to the US are usually employed in jobs that are physically and psychologically draining [33]. Immigrant women working abroad are more likely to suffer from physical injuries, physical and mental health problems when compared to immigrant men [32]. Women must also resolve work-family conflict whenever their work and schedule interfere with family or vice-versa [34]. Further research is necessary to understand how immigrant Latinas’ balance external work and familial obligations while also caring for themselves physically and emotionally [35].

Together, the codes identified in the socioecological context of Latina immigrant and Mexican women demonstrate they recognized the benefits that being physically active introduces to their health and want to be physically active, but face systemic, cultural, structural and social barriers that limit their ability to engage in physical activity. The barriers faced by women in both the US and Mexico can help explain the low physical activity rates reported among women in both countries, the sex differences, and the disproportional burden of chronic diseases experienced by Latina immigrant women in the US, and women in Mexico.

Certain limitations can be found throughout the study. The US sample had an overrepresentation of Mexican participants; most participants had low-income backgrounds and resided in small cities or rural towns. Thus, this study’s results cannot be generalized to multiple subgroups of Latino immigrant populations who reside in metropolitan areas and/or are of middle- or upper-class backgrounds. Additionally, another limitation is that the data collection occurred between February and April 2016 (7 years ago). The time gap between data collection and the present (2023) raises the possibility that certain factors and circumstances may have changed, potentially impacting the current relevance of the findings. Perceptions of physical activity may have changed, particularly during and after the Covid-19 pandemic experience. A final limitation of this study is the use of snowball sampling, which may have introduced selection bias. Although it has the potential to introduce selection biases, we selected a snowball sampling strategy to address the small size of the Latinx community and pre-existing trust issues. In this context, obtaining referrals from friends was a more effective approach for participant recruitment.

## Conclusion

Using a social-ecological framework to examine data from a transnational study, facilitators and barriers to physical activity in Latina immigrant women and Mexican women residing in the US and Mexico were elucidated. The findings suggest that Latina immigrant mothers and Mexican mothers face significant barriers that limit their ability to engage in physical activity. Beyond the shared barriers, our findings showed Latina immigrant mothers face additional environmental barriers upon arrival to the US. Despite the multilevel challenges, Latina immigrant mothers and Mexican mothers shared an interest in engaging in physical activity.

Efforts to increase physical activity levels among Latina immigrant and Mexican mothers would benefit from culturally relevant, family-based approaches and accessible information about physical activity opportunities and attainable goals. The transnational shared barriers to physical activity we identified, introduce an opportunity to develop binational policies and public health interventions to address mothers’ shared needs while also offering options tailored to respond to their specific environmental barriers. In particular, binational physical activity promotion approaches should consider ensuring access to affordable indoor facilities, elucidating opportunities to enhance walkability within their communities, and recognizing that small and unstructured improvements in physical activity yield important health benefits. By incorporating these recommendations, transnational interventions and initiatives can support both Latina immigrant and Mexican mothers in their pursuit of active lifestyles.

## Supporting information

S1 Checklist COREQ (COnsolidated criteria for REporting Qualitative research) checklist. (PDF)

S1 File Inclusivity in global research. (DOCX)
