# Correction: Barriers and facilitators of physical activity among Latina immigrant and Mexican mothers living in the US and Mexico: A qualitative study

**DOI:** 10.1371/journal.pone.0315958

**Published:** 2024-12-12

**Authors:** Nancy Jacquelyn Pérez-Flores, María Pineros-Leano, Katherine Damian, Ashley M. Toney, Liliana Aguayo

The following information is missing from the Funding statement: Publication costs were sponsored by the Emory Global Diabetes Research Center (EGDRC) under award 5P30DK111024-05, and Emory University’s Open Access Publishing Fund.
